# Automated recognition of chromosome fusion using an alignment-free natural vector method

**DOI:** 10.3389/fgene.2024.1364951

**Published:** 2024-03-20

**Authors:** Hongyu Yu, Stephen S.-T. Yau

**Affiliations:** ^1^ Department of Mathematical Sciences, Tsinghua University, Beijing, China; ^2^ Yanqi Lake Beijing Institute of Mathematical Science and Applications (BIMSA), Beijing, China

**Keywords:** chromosomal fusion, alignment-free, natural vector, Kuhn-Munkres algorithm, automated recognition

## Abstract

Chromosomal fusion is a significant form of structural variation, but research into algorithms for its identification has been limited. Most existing methods rely on synteny analysis, which necessitates manual annotations and always involves inefficient sequence alignments. In this paper, we present a novel alignment-free algorithm for chromosomal fusion recognition. Our method transforms the problem into a series of assignment problems using natural vectors and efficiently solves them with the Kuhn-Munkres algorithm. When applied to the human/gorilla and swamp buffalo/river buffalo datasets, our algorithm successfully and efficiently identifies chromosomal fusion events. Notably, our approach offers several advantages, including higher processing speeds by eliminating time-consuming alignments and removing the need for manual annotations. By an alignment-free perspective, our algorithm initially considers entire chromosomes instead of fragments to identify chromosomal structural variations, offering substantial potential to advance research in this field.

## 1 Introduction

**FIGURE 1:**
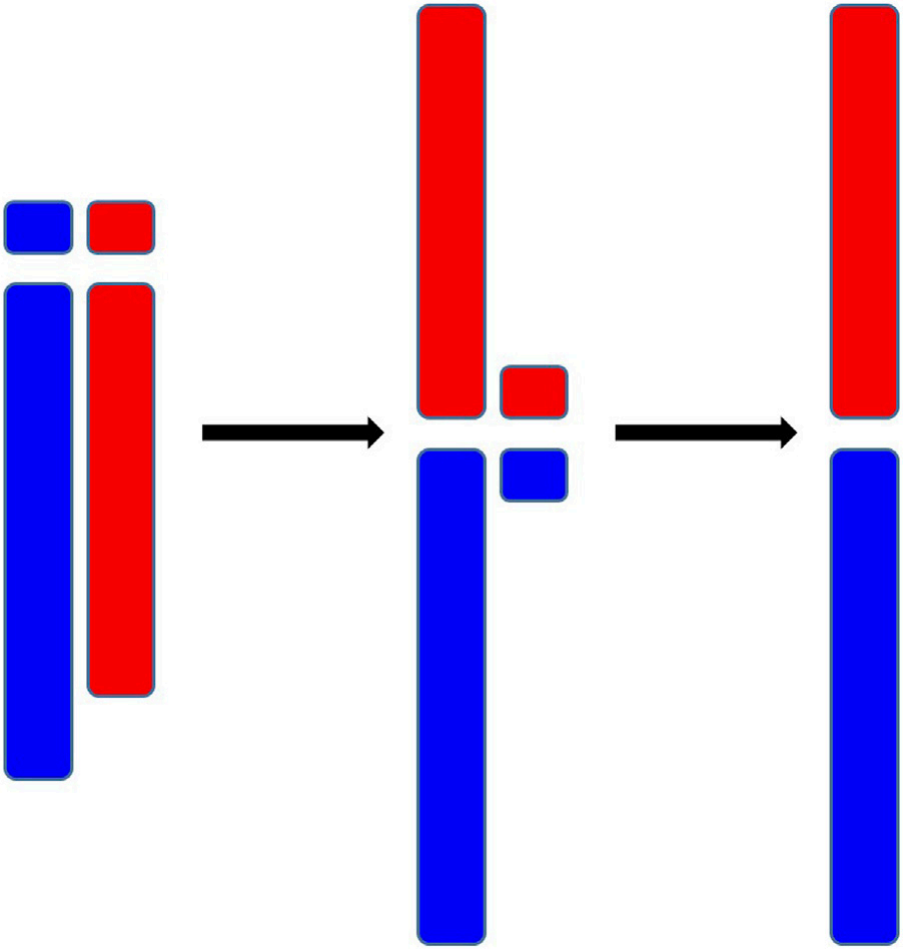
Demonstration of Robertsonian translocations.

Chromosome fusion, a genetic event wherein two or more separate chromosomes merge to form a single chromosome, is a substantial restructuring of the genome. A primary factor leading to the chromosome fusion is Robertsonian translocation, a chromosomal abnormality where the long arms of two different chromosomes are linked Poot and Hochstenbach (2021). As is shown in Figure 1, initially, the short arms of these two chromosomes are also linked, but they are usually lost afterward. (The short arms, being too short to harbor significant genetic information, may not lead to lethality upon their loss.) Chromosome fusion holds significant implications for cellular processes and biological evolution Vara et al. (2021); Feulner and De-Kayne (2017). On one hand, chromosome fusion can interfere the process of meiosis and lead to the production of imbalanced gametes, potentially diminishing reproductive compatibility Cicconardi et al. (2021); Hauffe and Searle (1998). On the other hand, chromosome fusion physically connects genes that were originally located on different chromosomes, thereby reducing their potential for recombination, which can be instrumental in preserving co-adapted alleles Cicconardi et al. (2021); Guerrero and Kirkpatrick (2014). These two facets underscore the significance of chromosome fusion in the process of speciation.

Chromosome fusion presents several well-documented instances across different species. One of the most prominent examples is the case of human chromosome 2. Humans possess 23 pairs of chromosomes, while other great apes, such as chimpanzees and gorillas, have 24 pairs. Extensive research suggests that human chromosome 2 is the result of the fusion of two ancestral chromosomes from great apes Yunis and OM (1982); Ijdo et al. (1991). Additionally, chromosome fusion can also occur within the same species. For instance, the water buffalo (Bubalus bubalis) consists of two distinct subspecies, the swamp buffalo and the river buffalo, with chromosome numbers of 48 and 50, respectively Iannuzzi et al. (2021). This difference in chromosome number is also attributed to chromosome fusion events.

Despite the significance of chromosome fusion as a structural variation in chromosomes, research in this domain has been relatively limited in comparison to gene-level investigations. Currently, there are several structural variation detection algorithms designed for identifying gene structural variations within the same species. These algorithms prove effective in detecting genetic diseases caused by structural variations in humans Cameron et al. (2021); Layer et al. (2014). However, their applicability between different species is challenging. For fusion events between different species, the mainstream methods center around synteny analysis, with examples including Fish, Cinteny, and MCScan Calabrese et al. (2003); Sinha and Meller (2007); Tang et al. (2008b,a); Wang et al. (2012). The fundamental approach of these algorithms can be summarized in two main steps. Firstly, chromosomes are partitioned into multiple regions utilizing experimentally obtained annotation information, such as the coding sequence range. Subsequently, alignment algorithms are applied to compare and analyze these regions Altschul et al. (1990); Haas et al. (2005). These algorithms provide the advantage of delivering clear and visually interpretable results. Nevertheless, they come with limitations, given their reliance on manual annotation as well as alignment algorithms which can be computationally intensive.

Indeed, methods exist for detecting structural variations in the human genome without relying on alignment Liu et al. (2021). However, these methods still involve partitioning the sequence into multiple regions and using strategies like k-mer search as a substitute for alignment, which is logically similar to alignment. We aim to adopt a more purely alignment-free perspective by directly embedding each sequence into a vector and performing operations solely on vectors, rather than comparing sequences with each other. There are many methods that map sequences into vectors Qi et al. (2004); Jun et al. (2009), and the natural vector method is an effective approach among them Deng et al. (2011); Wen et al. (2014b). By incorporating statistical moments, the natural vector works well in sequence comparison and phylogenetic analysis. Additionally, the convex hulls formed by natural vectors from distinct families do not overlap, demonstrating the favorable separation properties of natural vectors Wen et al. (2014a); Sun et al. (2021); Tian et al. (2018).

In this paper, we addressed the issue of chromosome fusion recognition from a novel perspective. We utilized the natural vector approach to extract statistical information from sets of chromosomes. Subsequently, we framed the pairing of chromosome sets as an assignment problem and identified the most likely fusion scenarios by minimizing the assignment loss Kuhn (1955). Applying this algorithm to the human/gorilla and swamp buffalo/river buffalo datasets, we successfully and efficiently identified the correct chromosome fusion scenarios without the need for annotations or alignments. Moreover, the process is significantly faster than traditional synteny analysis.

## 2 Materials and methods

### 2.1 Materials

The data utilized in this paper comprises the reference chromosomes of four distinct species: human (*Homo sapiens*), gorilla (*Gorilla gorilla*), swamp buffalo (Bubalus carabanensis), and river buffalo (Bubalus Bubalis). We downloaded these sequences from the National Center for Biotechnology Information (NCBI) on 10 October 2023. The sequences can be accessed through the following URL:https://ftp.ncbi.nlm.nih.gov/genomes/refseq/vertebrate_mammalian/.

The IDs of these sequences will be listed in the Supplementary Material S1, and hereafter, we use numerical labels to represent these chromosomes. The autosomes for humans, swamp buffalo, and river buffalo are numbered from 1 to 22, 1 to 23, and 1 to 24, respectively, while gorilla autosomes are numbered from 1, 2A, 2B, 3 to 22, consistent with the sequence annotation labels.

### 2.2 Problem formulation

We will first elaborate on the specific representation of the chromosome fusion problem. For convenience, we only considered the scenario where only a pair of chromosomes fused. The situations involving multiple fusion or the fusion of multiple chromosomes are similar and only require an expansion of the enumerated cases. Then, the task of recognizing chromosome fusion can be precisely described as follows: given two sets of chromosomes, *A* = {*a*_1_, *a*_2_, …, *a*_*n*_} and *B* = {*b*_1_, *b*_2_, …, *b*_*n*_, *b*_*n*+1_}, it is known that a pair of chromosomes from *B* fuse to form a single chromosome in *A*. The objective is to identify which pair of chromosomes from *B* fuse together and establish a correspondence between the remaining chromosomes in both sets.

Instead of fragmenting chromosomes and employing local alignment techniques as done in synteny analysis, our approach takes a holistic approach to analyze sequences from a different perspective. In the subsequent sections, we will first introduce the extraction of sequence information using *k*-mer natural vectors. Following that, we will discuss the assignment problem and its corresponding solution, the Kuhn-Munkres algorithm. Finally, we will illustrate how to transform the chromosome fusion problem into an assignment problem using *k*-mer natural vectors and subsequently solve it.

### 2.3 Natural vectors and their properties in chromosome fusion

The natural vector method is an alignment-free technique that converts DNA sequences into moment vectors Deng et al. (2011). For a given DNA sequence *S* = *s*_1_ *s*_2_…*s*_*n*_, we define:

$$w_{k}\left(s_{i}\right)=\left\{\begin{array}{c} 1,\;s_{i}=k\;\\ 0,\;otherwise\; \end{array}\right.$$ (1)

where *k*, *s*_*i*_ ∈ {*A*, *T*, *C*, *G*}. Then the order 2 natural vector *nv*(*S*) can be defined as

$$nv\left(S\right)=\left(n_{A},n_{C},n_{G},n_{T},\mu_{A},\mu_{C},\mu_{G},\mu_{T},D_{2}^{A},D_{2}^{C},D_{2}^{G},D_{2}^{T}\right)$$ (2)

where

$$\left\{\begin{array}{c} n_{k}=\overset{N}{\underset{i=1}{\sum }}w_{k}\left(s_{i}\right)\;\\ \mu_{k}=\overset{N}{\underset{i=1}{\sum }}\frac{i}{n_{k}}w_{k}\left(s_{i}\right)\;\\ D_{2}^{k}=\overset{N}{\underset{i=1}{\sum }}\frac{{\left(i-\mu_{k}\right)}^{2}}{n_{k}N}w_{k}\left(s_{i}\right)\;\\ N=n_{A}+n_{T}+n_{C}+n_{G}\; \end{array}\right.$$ (3)

If *n*_*k*_ = 0, we let $\mu_{k}=D_{2}^{k}=0$ . In this paper, we concentrate on only the order 2 natural vectors; thus, we will omit the order designation in the following content.

Given two single-stranded sequences, *S*_1_ and *S*_2_, which are oriented from 5′ to 3’, there are two possible representations of fusion, denoted as *S*_1_ + *S*_2_ (with *S*_1_ in the front) and *S*_2_ + *S*_1_ (with *S*_2_ in the front). However, for double-stranded sequences, it becomes much more complex. Each chromosome consists of two strands named the forward strand and the reverse strand respectively. Consequently, there are a total of 8 possible representations of fusion. If the number of segments increases to *k*, the number of possible representations of fusion increases rapidly to *k*! × 2^*k*^. Therefore, it is not efficient to generate each possible fusion and calculate their natural vectors separately. In fact, if we have already calculated *nv* (*S*_1_) and *nv* (*S*_2_), we can obtain *nv* (*S*_3_) where *S*_3_ = *S*_1_ + *S*_2_ through the following calculation.Let $nv\left(S_{i}\right)=\left(n_{iA},n_{iC},n_{iG},n_{iT},\mu_{iA},\mu_{iC},\mu_{iG},\mu_{iT},D_{2}^{iA},D_{2}^{iC},D_{2}^{iG},D_{2}^{iT}\right)$ , *N*_*i*_ = *n*_*iA*_ + *n*_*iC*_ + *n*_*iG*_ + *n*_*iT*_, and *C*(*S*, *k*) = {*i*|*w*_*k*_ (*s*_*i*_) = 1} where *S* = *s*_1_ *s*_2_…*s*_*n*_, then we have

$$\begin{array}{ll} n_{3k}= & n_{1k}+n_{2k}\\ \mu_{1k}= & \frac{\underset{i\in C\left(S_{1},k\right)}{\sum }i}{n_{1k}}\\ \mu_{2k}= & \frac{\underset{i\in C\left(S_{2},k\right)}{\sum }i}{n_{2k}} \end{array}$$ (4)

$$\begin{array}{ll} \mu_{3k}= & \frac{\underset{i\in C\left(S_{1},k\right)}{\sum }i+\underset{i\in C\left(S_{2},k\right)}{\sum }\left(i+N_{1}\right)}{n_{1k}+n_{2k}}\\ = & \frac{n_{1k}}{n_{1k}+n_{2k}}\mu_{1k}+\frac{n_{2k}}{n_{1k}+n_{2k}}\left(\mu_{2k}+N_{1}\right) \end{array}$$ (5)

$$\begin{array}{ll} & \;\;\;n_{1k}N_{1}D_{2}^{1k}=\underset{i\in C\left(S_{1},k\right)}{\sum }{\left(i-\mu_{1k}\right)}^{2}\\ & \;\;\;n_{2k}N_{2}D_{2}^{2k}=\underset{i\in C\left(S_{2},k\right)}{\sum }{\left(i-\mu_{2k}\right)}^{2}\\ & \underset{i\in C\left(S_{1},k\right)}{\sum }{\left(i-\mu_{3k}\right)}^{2}+\underset{i\in C\left(S_{2},k\right)}{\sum }{\left(i+N_{1}-\mu_{3k}\right)}^{2}\\ = & \underset{i\in C\left(S_{1},k\right)}{\sum }{\left(i-\mu_{1k}\right)}^{2}+\underset{i\in C\left(S_{1},k\right)}{\sum }{\left(\mu_{1k}-\mu_{3k}\right)}^{2}\\ & +2\underset{i\in C\left(S_{1},k\right)}{\sum }\left(\mu_{1k}-\mu_{3k}\right)\left(i-\mu_{1k}\right)+\underset{i\in C\left(S_{2},k\right)}{\sum }{\left(i-\mu_{2k}\right)}^{2}\\ & +\underset{i\in C\left(S_{2},k\right)}{\sum }{\left(\mu_{2k}+N_{1}-\mu_{3k}\right)}^{2}+2\underset{i\in C\left(S_{2},k\right)}{\sum }\left(\mu_{2k}+N-\mu_{3k}\right)\left(i-\mu_{2k}\right)\\ = & \underset{i\in C\left(S_{1},k\right)}{\sum }{\left(i-\mu_{1k}\right)}^{2}+\underset{i\in C\left(S_{2},k\right)}{\sum }{\left(i-\mu_{2k}\right)}^{2}\\ & +n_{1k}{\left(\mu_{1k}-\mu_{3k}\right)}^{2}+n_{2k}{\left(\mu_{2k}+N_{1}-\mu_{3k}\right)}^{2} \end{array}$$ (6)

$$\begin{array}{ll} D_{2}^{3k}= & \frac{\underset{i\in C\left(S_{1},k\right)}{\sum }{\left(i-\mu_{3k}\right)}^{2}+\underset{i\in C\left(S_{2},k\right)}{\sum }{\left(i+N_{1}-\mu_{3k}\right)}^{2}}{\left(N_{1}+N_{2}\right)\left(n_{1k}+n_{2k}\right)}\\ = & \frac{n_{1k}N_{1}D_{2}^{1k}+n_{2k}N_{2}D_{2}^{2k}+n_{1k}{\left(\mu_{1k}-\mu_{3k}\right)}^{2}+n_{2k}{\left(\mu_{2k}+N_{1}-\mu_{3k}\right)}^{2}}{\left(N_{1}+N_{2}\right)\left(n_{1k}+n_{2k}\right)} \end{array}$$ (7)

Also, Given a vector of the forward strand

$$nv\left(S\right)=\left(n_{A},n_{C},n_{G},n_{T},\mu_{A},\mu_{C},\mu_{G},\mu_{T},D_{2}^{A},D_{2}^{C},D_{2}^{G},D_{2}^{T}\right)$$

and *N* = *n*_*A*_ + *n*_*T*_ + *n*_*C*_ + *n*_*G*_, the vector of the corresponding reverse strand *R*(*S*) can be represented as

$$\begin{array}{lll} nv\left(R\left(S\right)\right)= & \left(n_{T},n_{G},n_{C},n_{A},\right.N+1-\mu_{T}, & \left.N+1-\mu_{G},N+1-\mu_{C},N+1-\mu_{A},D_{2}^{T},D_{2}^{G},D_{2}^{C},D_{2}^{A}\right). \end{array}$$ (8)

It is worth noticing that we should not only take the complementary chain but also reverse it since the orientation of two strands is opposite.

### 2.4 K-mer natural vectors and their properties in chromosome fusion

The *k*-mer natural vector method is an extension of the natural vector method which considers *k*-mers instead of nucleotides as basic elements in sequences Wen et al. (2014b); Sun et al. (2021). *K*-mer is a string composed of *k* nucleotides and there are 4^*k*^ possible *k*-mers (denoted by $l_{1},\ldots ,l_{4^{k}}$ ). For the sequence *S* = *s*_1_ *s*_2_…*s*_*n*_, we can regard it as a sequence consisting of *n* − *k* + 1 *k*-mers (*s*_1_…*s*_*k*_)…(*s*_*n*−*k*+1_…*s*_*n*_). Similar to traditional natural vectors, we can define the *k*-mer natural vector

$$nv_{k}\left(S\right)=\left(n_{l_{1}},\ldots ,n_{l_{k}},\mu_{l_{1}},\ldots ,\mu_{l_{k}},D_{2}^{l_{1}},\ldots ,D_{2}^{l_{k}}\right).$$

If $n_{l_{i}}=0$ , we let $\mu_{l_{i}}=D_{2}^{l_{i}}=0$ . In the case of traditional natural vectors, we can compute *nv*_*k*_ (*S*_3_) (where *S*_3_ = *S*_1_ + *S*_2_) using *nv*_*k*_ (*S*_1_) and *nv*_*k*_ (*S*_2_). When dealing with *k*-mer natural vectors, we can follow a similar process, but it’s important to note that the results obtained from formulas (5) and (7) are no longer exact but approximate.

Let’s take a 2-mer example for clarity. If we have the sequence *S*_1_ = *s*_1_ *s*_2_…*s*_*n*_ and the sequence *S*_2_ = *t*_1_ *t*_2_…*t*_*n*_, we can consider them as (*s*_1_ *s*_2_)…(*s*_*n*−1_ *s*_*n*_) and (*t*_1_ *t*_2_)…(*t*_*n*−1_ *t*_*n*_), respectively. The results obtained from formulas (5) and (7) represent the natural vector of (*s*_1_ *s*_2_)…(*s*_*n*−1_ *s*_*n*_)(*t*_1_ *t*_2_)…(*t*_*n*−1_ *t*_*n*_) instead of (*s*_1_ *s*_2_)…(*s*_*n*−1_ *s*_*n*_)(*s*_*n*_ *t*_1_)(*t*_1_ *t*_2_)…(*t*_*n*−1_ *t*_*n*_) that corresponds to *S*_1_ + *S*_2_. However, given the considerable length of chromosomes, the difference introduced by a single *k*-mer becomes negligible. Therefore, we can still apply the previous formulas to perform the calculations effectively.

Previous studies have indicated that the optimal value for *k* should fall within the range of [*ceil* (*log*_4_ *min* (*LS*)), *ceil* (*log*_4_ *max* (*LS*)) + 1] where *LS* represents the set of lengths of genetic sequences in the study Wen et al. (2014b). For the datasets under consideration, the optimal *k* to extract the information of the sequences ranges from 13 to 15. However, this *k* is excessively large and does not fully leverage the time complexity advantage of our algorithm. Therefore, in this paper, we set *k* = 10, the smallest *k* to ensure that the algorithm avoids errors on our datasets.

### 2.5 The assignment problem and the Kuhn-Munkres algorithm

An assignment problem represents a specific instance of the more general transportation problem. In this particular case, the goal is to assign a set of resources to an equal number of activities while minimizing the total cost or maximizing the total profit of the allocation. To elaborate further, the problem can be formally stated as follows: given an *n* × *n* matrix *M* = (*m*_*ij*_), we aim to determine an optimal permutation *p*_1_, *p*_2_, …, *p*_*n*_ from the set 1, 2, …, *n* in order to minimize or maximize the objective function $\overset{n}{\underset{i=1}{\sum }}m_{ip_{i}}$ .

Simply enumerating all possible permutations is feasible for small values of *n*. However, for larger values of *n*, this approach becomes computationally expensive and impractical as there are *n*! possible permutations. In such cases, the Kuhn-Munkres algorithm, also known as the Hungarian method, offers an efficient solution to this problem Kuhn (1955, 1956); Munkres (1957).

The Kuhn-Munkres algorithm is a combinatorial optimization algorithm that can solve the assignment problem in polynomial time. The original version of the algorithm has a time complexity of *O* (*n* ^4^), but later improvements have reduced it to *O* (*n* ^3^) Edmonds and Karp (2003); Tomizawa (1971).

In Algorithm 1, we demonstrate how to minimize the objective function given matrix *M* by the Kuhn-Munkres algorithm:

Algorithm 1. The Kuhn-Munkres algorithm.

Subtract the minimum entry in each row from all other entries in the same row.
Subtract the minimum entry in each column from all other entries in the same column.
**while** There are no *m* lines (rows or columns) that cover all zeros, where *m* < *n* **do**
Find the minimum entry not covered by any line and its value is *e*.
Subtract *e* from each uncovered row and add *e* to each covered column.
**end while**
We can select *n* zeros with distinct rows and columns, which corresponds to the optimal choice.

### 2.6 The algorithm to recognize chromosome fusion

In order to transform the chromosome fusion problem into an assignment problem, we need to define a good measure for the similarity between chromosomes. One straightforward approach is to employ the Euclidean distance between the *k*-mer natural vectors of the chromosomes. However, sequencing chromosomes can introduce substantial errors and lead to length variation. Directly using the Euclidean distance might be problematic as the Euclidean distance between natural vectors is length-sensitive which could amplify the errors. To mitigate the effects of sequence length variations, we propose two distinct measures to dissociate the impact of the length from the *k*-mer patterns:

$$D_{1}\left(a,b\right)=1-\max\left(\cos∠\left(nv_{K}\left(a\right),nv_{K}\left(b\right)\right),\cos∠\left(nv_{K}\left(a\right),nv_{K}\left(R\left(b\right)\right)\right)\right)$$ (9)

$$D_{2}\left(a,b\right)=|\text{length}\left(a\right)-\text{length}\left(b\right)|$$ (10)

In the above equations, *∠*(., .) represents the angle between the two vectors. In *D*_1_, we need to take both two strands into account so *R*(*b*) should also be considered.

It is worth noticing that formula (9) will be slightly modified for fused chromosomes because there are eight possible representations for fused chromosomes, as opposed to the two representations for normal chromosomes. Assuming the fused chromosome is ${\tilde{b}}_{1}$ from the set *v*_1_, …, *v*_8_, then formula (9) is adjusted as follows:

$$D_{1}\left(a_{i},{\tilde{b}}_{1}\right)=1-\underset{j=1,\ldots ,8}{\max}\left(\cos∠\left(nv_{K}\left(a_{i}\right),nv_{K}\left(v_{j}\right)\right)\right).$$ (11)

Given two sets of chromosomes of equal count, *A* = {*a*_1_, *a*_2_, …, *a*_*n*_} and $\tilde{B}=\left\{{\tilde{b}}_{1},{\tilde{b}}_{2},\ldots ,{\tilde{b}}_{n}\right\}$ . The correspondence between these sets can be denoted by (*p*_1_, …, *p*_*n*_), a permutation of {1, …, *n*}. That is, ${\tilde{b}}_{p_{i}}$ is similar to *a*_*i*_. The task of establishing chromosome correspondence can be formulated as an optimization problem:

$$L\left(A,\tilde{B}\right)=\underset{p_{1},\ldots ,p_{n}}{\min}\overset{n}{\underset{i=1}{\sum }}\left(\overset{2}{\underset{l=1}{\sum }}N\left(D_{l}\left(a_{i},{\tilde{b}}_{p_{i}}\right)|D_{l}\left(a_{i},.\right)\right)\right)$$ (12)

where

$$N\left(x|A\right)=\frac{x-min\left(A\right)}{max\left(A\right)-min\left(A\right)}$$ (13)

serves as a normalization function to balance the importance of *D*_1_ and *D*_2_ with different orders of magnitude.

Eq 12 can be comprehended from two perspectives. First, by solving this optimization problem with the help of the Kuhn-Munkres algorithm, we can determine the optimal correspondence between the two chromosome sets. Second, the defined function *L* can serve as a metric, indicating the closeness of the two chromosome sets. Considering various possible fusion that convert *B* = {*b*_1_, *b*_2_, …, *b*_*n*_, *b*_*n*+1_} into $\tilde{B}=\left\{{\tilde{b}}_{1},{\tilde{b}}_{2},\ldots ,{\tilde{b}}_{n}\right\}$ , the fusion resulting in the smallest loss function *L* would be our desired transformation.

To provide a clearer representation of the algorithm’s process, we briefly summarize it in the following Algorithm 2. The code can be found in https://github.com/BobYHY/Fusion/.

Algorithm 2. Chromosome Fusion Recognition Algorithm.

Calculate *k*-mer natural vectors for all chromosomes.
Calculate *D*_1_ and *D*_2_ for all pairs of chromosomes, including potential new ones resulting from fusion.
**for** *j*_1_ = 1 to *n* + 1 **do**
 **for** *j*_2_ = *j*_1_ + 1 to *n* + 1**do**
  Fuse $b_{j_{1}}$ and $b_{j_{2}}$ in eight possible ways (*v*_1_, *v*_2_, …, *v*_8_) and calculate their *k*-mer natural vectors using Eqs 5, 7. The resulting set after fusion is denoted as ${\tilde{B}}_{j_{1}j_{2}}$ .
  Calculate $L_{j_{1}j_{2}}=L\left(A,{\tilde{B}}_{j_{1}j_{2}}\right)$ using the Kuhn-Munkres algorithm given the precomputed *D*_1_ and *D*_2_.
 **end for**
**end for**
The smallest $L_{j_{1}j_{2}}$ corresponds to the most likely fusion.

Suppose each chromosome has a length of *O*(*l*), then the time complexity of the *k*-mer natural vector algorithm is *O* (*nl*). Next, measuring the distances between all pairs of chromosomes, including existing chromosomes and potentially new ones resulting from fusion, has a time complexity of *O* (*n* ^3^4^*k*^). The Kuhn-Munkres algorithm has a complexity of *O* (*n* ^3^), and it needs to be run for all possible fusion scenarios. Therefore, the overall complexity is *O* (*nl* + *n* ^3^4^*k*^ + *n* ^5^).

### 2.7 Multidimensional scaling

Multidimensional scaling (MDS) is a technique for visualizing the similarity between individual cases within a dataset Mead (1992). Its underlying concept is quite straightforward: how to find a set of points in a plane in such a way that the distances between them closely approximate a given distance matrix. More precisely, if we have a distance matrix *D* = (*d*_*ij*_) for *n* chromosomes and we wish to map them to positions *x*_1_, …, *x*_*n*_ on a plane, then MDS formulates an optimization problem to minimize the following expression:

$$f\left(x_{1},\ldots ,x_{n}\right)=\underset{i\neq j}{\sum }{\left(d_{ij}-\|x_{i}-x_{j}\|\right)}^{2}.$$ (14)

It is worth noting that in the earlier process of identifying chromosomal fusions, we did not calculate the distances within the same chromosome group. In fact, we can employ the normalized distances as defined in Eq. 12 for cases where *A* and *B* are the same sets, and then symmetrized the results to obtain the distances within the chromosome groups.

### 2.8 Synteny analysis

Synteny plots were generated using the MCScan module from the jcvi library Tang et al. (2008a). The data used for this analysis included chromosome sequences and GTF annotation data. The ‘minspan’ parameter was set to 50 to control the minimum span of syntenic blocks in the analysis. The synteny plot provides a visual representation of conserved gene order and genomic rearrangements between different species.

## 3 Results and discussion

### 3.1 Chromosome fusion recognition for human/gorilla and swamp buffalo/river buffalo

We employed our algorithm to identify chromosome fusion in human/gorilla and swamp buffalo/river buffalo datasets. It’s worth noting that we did not include sex chromosomes in our analysis for two main reasons. First, the presence of palindromes in sex chromosomes, especially in Y chromosome, complicates its sequencing and results in a higher error rate compared to other chromosomes Trombetta and Cruciani (2017). Second, identifying sex chromosomes in the XY pair is straightforward due to their unequal lengths, obviating the need for explicit matching.

The results of our algorithm reveal that the gorilla chromosomes 2A and chromosome 2B have fused into a single sequence, aligning with human chromosome 2. Additionally, the river buffalo chromosome 4 and chromosome nine have fused into a single sequence, aligning with the swamp buffalo chromosome 1. This outcome is consistent with the data annotations. After identifying the correct fusion scenarios, all chromosomes can also find their corresponding chromosomes in the other set.

**FIGURE 2:**
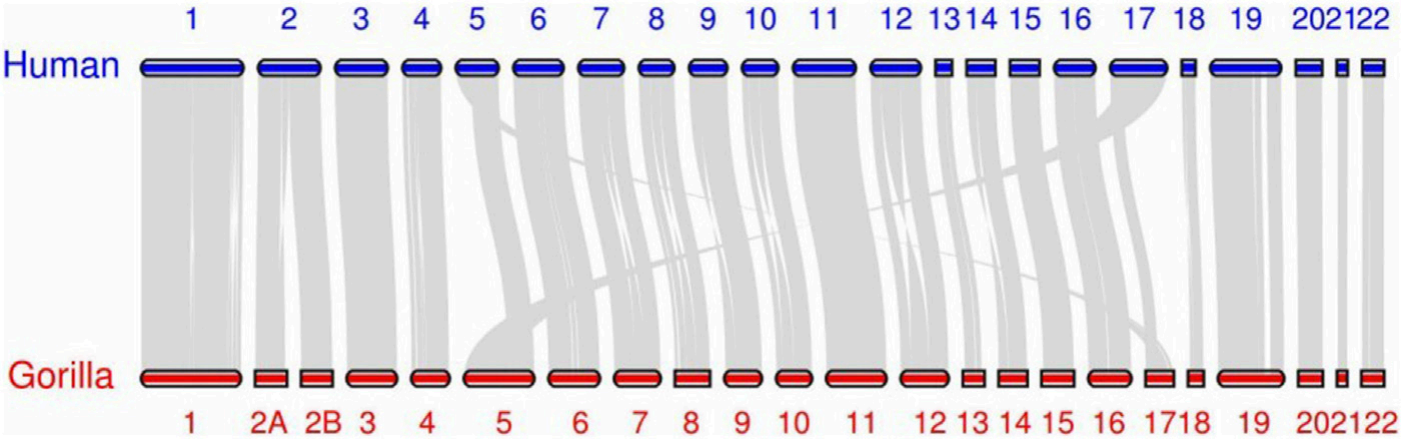
Synteny plots based on MCScan human/gorilla.

**FIGURE 3:**
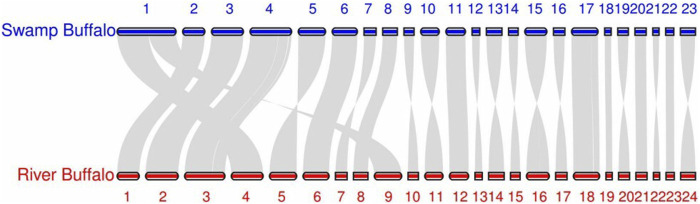
Synteny plots based on MCScan swamp buffalo/river buffalo.

It is worth noting that in our algorithm, chromosome pairing between the two sets is achieved globally by minimizing the total loss. This means that at the algorithmic level, we do not require the paired sequences to be each other’s nearest neighbors Cover and Hart (1967). (In this context, ‘near’ refers to a smaller pairing loss.) This design enhances the algorithm’s robustness, preventing scenarios where multiple sequences might share the same nearest neighbor due to other mutations, thus avoiding situations that could disrupt the one-to-one correspondence. However, in terms of the results, almost all pairings meet the nearest neighbor condition. All swamp buffalo chromosomes match their nearest neighbors, while all human chromosomes except one have their counterparts as nearest neighbors. The only exception is human chromosome 17, which has its corresponding counterpart as the second nearest neighbor. This exception aligns with the reality. Figures 2, 3 display the synteny analysis of chromosomes for human/gorilla and swamp buffalo/river buffalo using the MCScan method. It is evident that, in the case of human chromosome 17, due to some non-fusion structural variations, a portion of it actually originates from gorilla chromosome 5.

**FIGURE 4:**
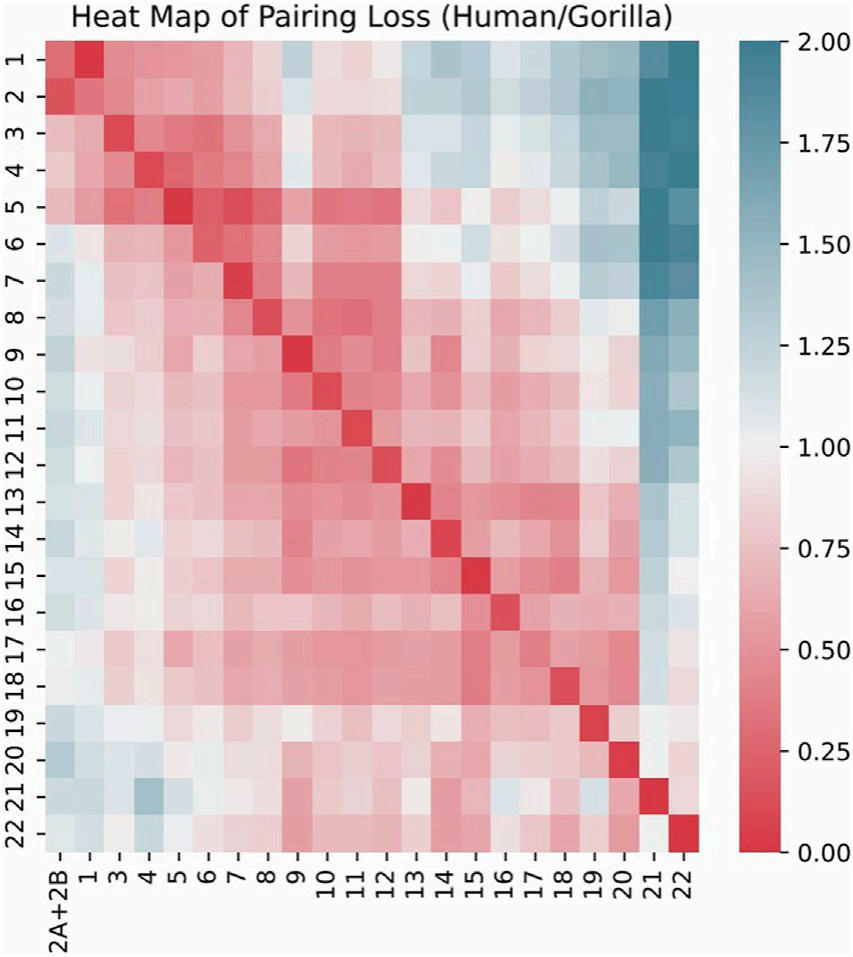
Heat map of pairing loss for chromosomes human/gorilla.

**FIGURE 5:**
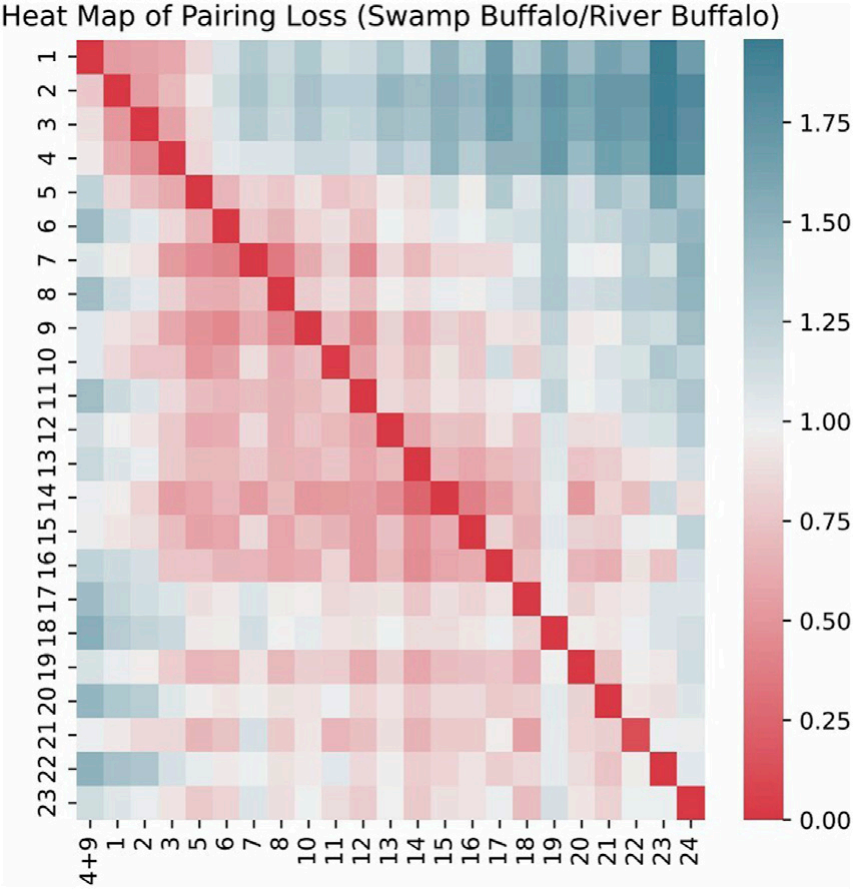
Heat map of pairing loss for chromosomes swamp buffalo/river buffalo.

**FIGURE 6:**
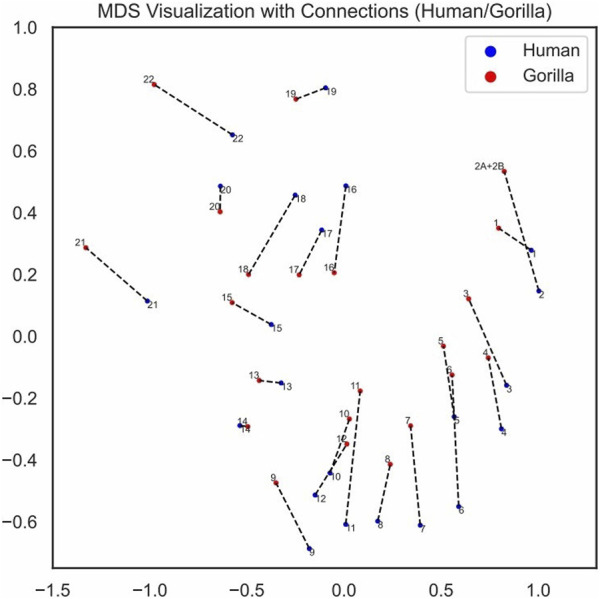
MDS visualization of chromosomes with pairing connections human/gorilla.

**FIGURE 7:**
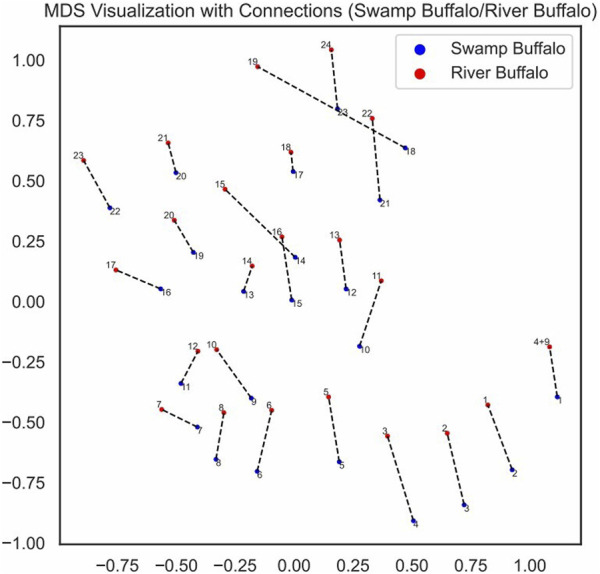
MDS visualization of chromosomes with pairing connections swamp buffalo/river buffalo.

We visualized the pairing loss between chromosomes in two sets after fusion using two different approaches. In Figures 4, 5, we employed the conventional heatmap representation. The smaller the pairing loss, the redder the corresponding square. In Figures 6, 7, we used multidimensional scaling (MDS) to project chromosomes as points onto a two-dimensional plane. The distances in the image can to some extent reflect the magnitude of the pairing loss. This representation offers greater intuitiveness, yet it’s important to note that, since MDS involves the projection of high-dimensional information onto a two-dimensional plane, the distances in the graph may contain discrepancies compared to actual distances. Employing both of these methods effectively demonstrates our ability to accurately measure the differences between chromosomes using alignment-free features.

### 3.2 Chromosome fusion recognition for synthetic fusion scenarios

In order to further demonstrate the effectiveness of our method, we conducted recognition in synthetic fusion scenarios. Specifically, we transformed the swamp buffalo/river buffalo dataset to generate a large number of fusion scenarios for testing. Initially, we fused chromosome 4 and chromosome nine of river buffalo in the correct manner. Subsequently, we artificially fused two random chromosomes from swamp buffalo, resulting in a new scenario where river buffalo still has one more chromosome than swamp buffalo.

Given that swamp buffalo has 23 autosomes, we obtained 253 possible fusion scenarios. (Different orientations and fusion orders are algorithmically equivalent.) We conducted chromosome fusion recognition on each of these 253 scenarios, successfully identifying the fusion pairing and accurately correlating the remaining chromosomes 252 times. In other words, our method achieved an accuracy rate of 99.6%, demonstrating its high performance.

### 3.3 Effectiveness and efficiency of the algorithm

Due to the fact that a chromosome has two complementary strands, when sequencing closely related species, we cannot ensure that the corresponding chromosome strands are the same. This is precisely the case with the swamp buffalo/river buffalo dataset. Therefore, our algorithm incorporates the consideration of complementary strands. When computing the pairing loss, we opt for the strand with the minimal loss to address this issue.

We validated the effectiveness of our algorithm through two real fusion scenarios and 253 synthetic fusion scenarios. Among all scenarios, we encountered errors in only one synthetic case, showcasing an impressive level of accuracy.

**FIGURE 8:**
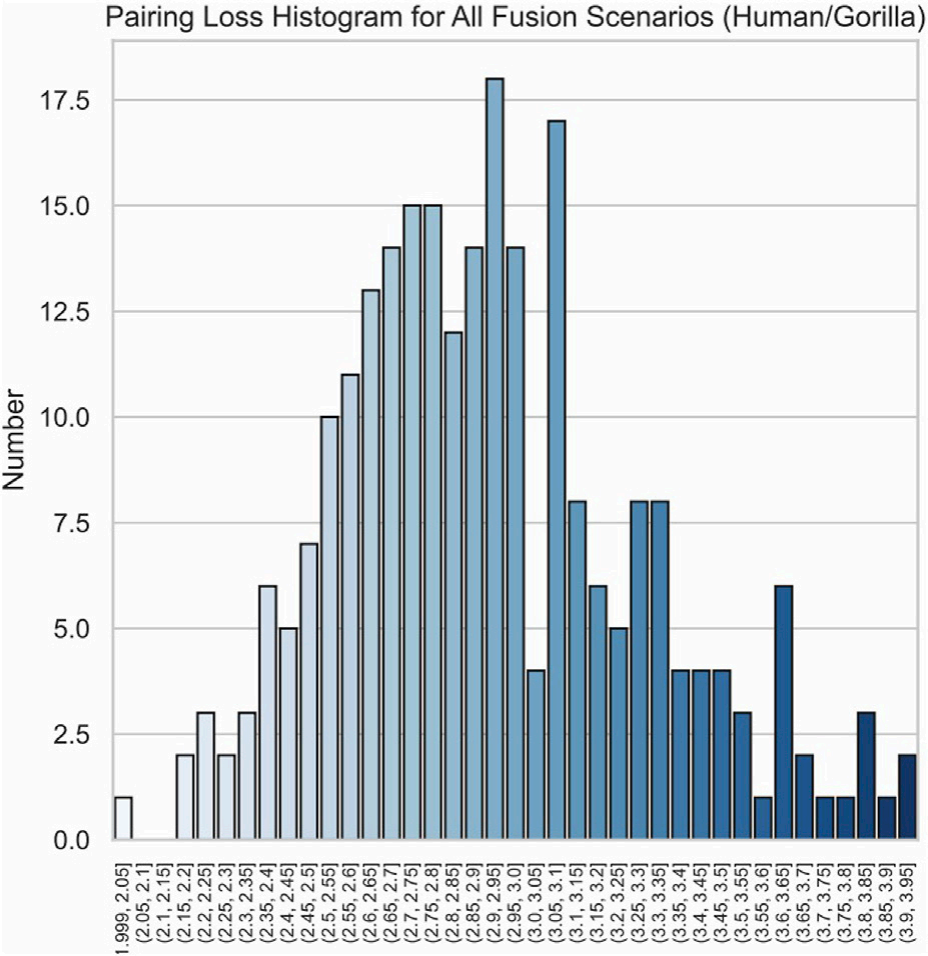
Histograms of pairing loss for all possible fusion scenarios human/gorilla.

**FIGURE 9:**
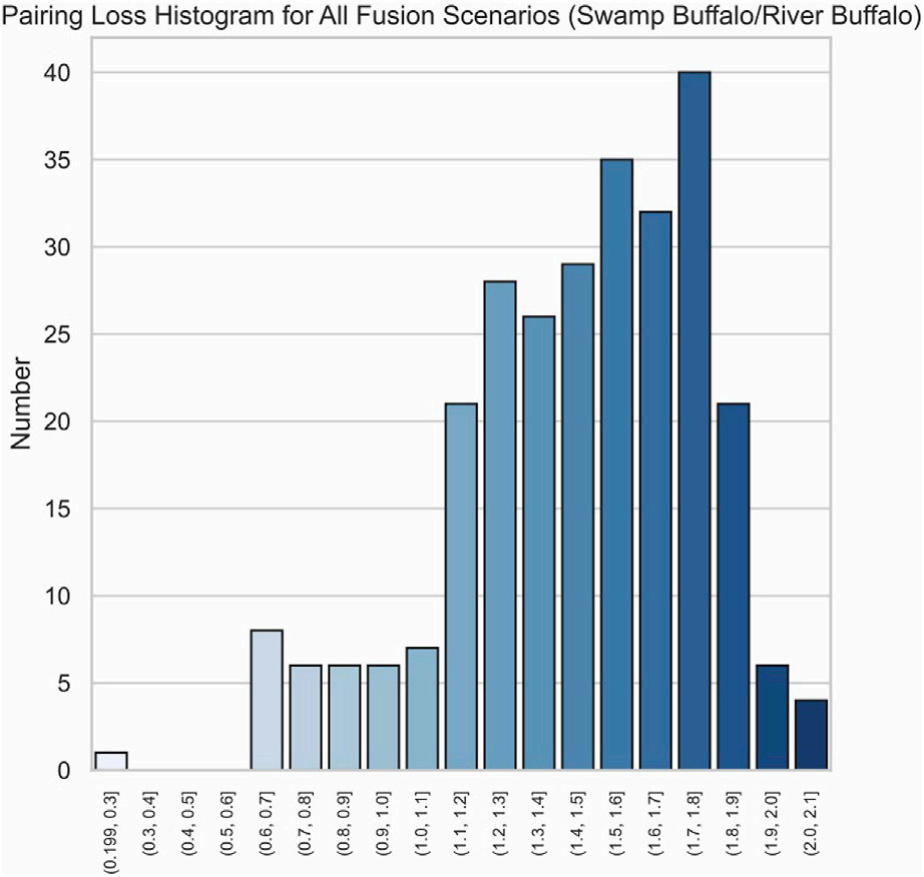
Histograms of pairing loss for all possible fusion scenarios swamp buffalo/river buffalo.

**FIGURE 10:**
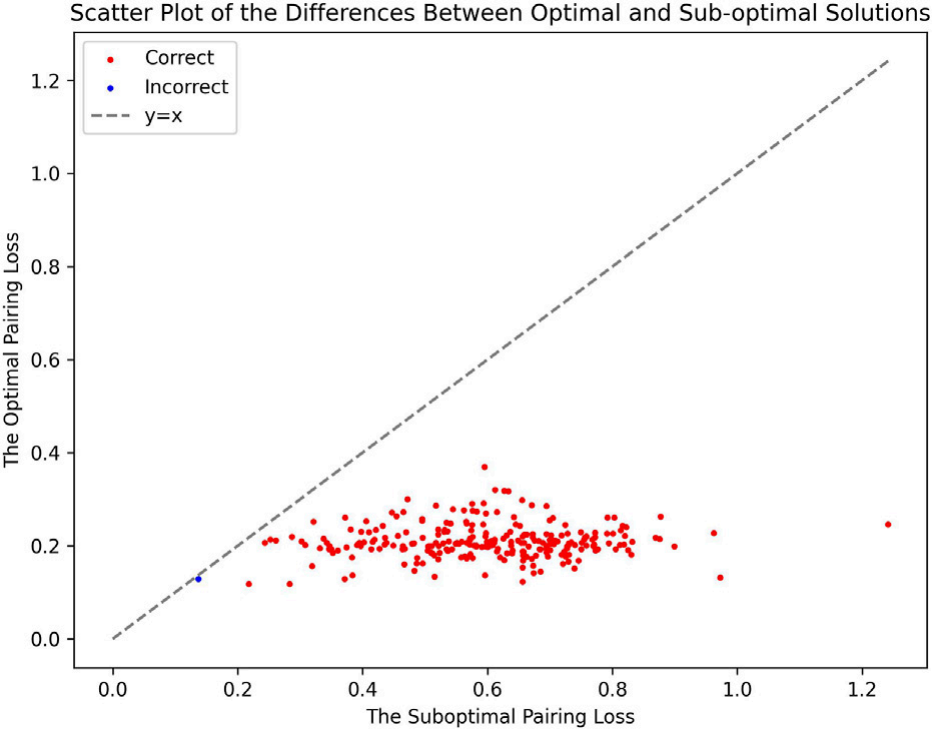
Scatter plot of the differences between optimal and sub-optimal solutions.

The effectiveness of our method is further highlighted by the pronounced characteristics of the correct fusion scenarios. In real fusion, we observed that in the case of human/gorilla, the pairing loss for the optimal fusion is 2.03, whereas the pairing losses for the other 252 possible fusions range from 2.16 to 3.92. Notably, the average difference in pairing loss for non-optimal fusions is 7.0 × 10^−3^, significantly smaller than the 0.13 difference between the optimal and sub-optimal fusions (Figure 8). Similarly, for the swamp buffalo/river buffalo dataset, the optimal fusion has a pairing loss of 0.26, while the pairing losses for the other 275 possible fusions range from 0.61 to 2.06. The average difference in pairing loss for non-optimal fusion is 5.3 × 10^−3^, again significantly smaller than the 0.35 difference between the optimal and sub-optimal fusions (Figure 9). In Figure 10, we can also observe that in synthetic fusion scenarios, the optimal pairing loss are consistently much smaller than the sub-optimal solutions. The only exception where the optimal and sub-optimal pairing loss are relatively close is the case of the error, as previously mentioned.

From the above results, we can draw two conclusions. Firstly, in both real fusion scenarios and synthetic fusion scenarios, the computed optimal loss is significantly smaller than the sub-optimal loss. This reflects the robustness of the algorithm, meaning that perturbing the original data won’t immediately change the optimal solution. This underscores the recognizability of the features associated with the correct fusion event. Secondly, the gap between the optimal and sub-optimal solutions can reflect the reliability of the results. Generally, correct identification is usually associated with a significant difference between these two values. Conversely, if they are very close, it may indicate potential issues with the results. Additionally, it can be observed that, compared to the recognition between the two types of buffalo, the identification of human/gorilla is relatively less reliable. This is attributed to the presence of another significant chromosomal structural variation in this example, namely, the exchange between chromosome five and chromosome 17 (Figure 2).

This algorithm does not require alignment and is therefore faster than previous methods. In real fusion scenarios, conducting synteny analysis for human/gorilla and swamp buffalo/river buffalo using MCScan took 1,052 and 993 s, respectively. Using our algorithm, computing *k*-mer natural vectors took 182 and 185 s respectively, and determining the most probable fusion scenarios with the algorithm took 271 and 293 s respectively (CPU 3.10GHz, 8C16T). (We use parallel computing to calculate each natural vector independently.) In synthetic fusion scenarios, we do not need to calculate natural vectors separately, and the average time spent on determining the most probable fusion scenarios is 265 s.

It’s worth mentioning that, in fact, we can disregard the time required for computing *k*-mer natural vectors. We can precompute the natural vectors and simply read them when comparing with other organisms. This is because if there are *M* organisms, the calculation of natural vectors only needs to be performed *O*(*M*) times. However, the comparisons require *O* (*M* ^2^) operations. Therefore, it is reasonable to focus solely on the fusion identification time. This fact can also be observed in synthetic scenarios, where natural vectors are all precomputed. Furthermore, even when including the time spent computing *k*-mer natural vectors, our algorithm remains faster.

Another noteworthy point is that in MCScan, our analysis is limited to experimentally determined CDS sequences, which account for only about 10% of the entire genome. This analysis relies heavily on manual experimental annotation and utilizes incomplete information. If one needs to segment the entire chromosome, the time required would significantly increase. In contrast, our method does not require annotation and allows for a rapid analysis of the entire chromosome.

## 4 Conclusion

In this study, we propose an alignment-free algorithm based on natural vectors and the Kuhn-Munkres algorithm to address the challenge of chromosome fusion recognition. Our approach offers a fresh perspective on understanding chromosome fusion phenomena. Previously, most alignment-free methods struggled to tackle the intricate issue of chromosome internal structures, while our method demonstrates significant improvements.

Our method has two main advantages. Firstly, our algorithm demonstrates a significant speed advantage, being about four times faster than synteny-based methods for datasets we consider. This allows for efficient data processing while maintaining high accuracy. Secondly, it considers whole chromosomes instead of segments, eliminating the need for manual selection of segment boundaries and additional annotation data, making the algorithm more automated.

However, our method still has limitations. It is primarily designed for fusion recognition and cannot detect other non-fusion structural variations, such as repeats. Additionally, in situations where multiple fusions occur, the speed advantages may diminish. In future studies, we aim to incorporate heuristic search designs to further enhance the algorithm’s speed, especially in scenarios involving multiple fusions, while maintaining the accuracy of the algorithm. Additionally, we aspire to identify alignment-free features for more localized chromosome structural variations.

## Data Availability

Publicly available datasets were analyzed in this study. This data can be found here: https://ftp.ncbi.nlm.nih.gov/genomes/refseq/vertebrate_mammalian/.

## Author’s Note

Dedicated to Dr. Henry Laufer on the occasion of his 80th birthday.
